# Application of Mesenchymal Stem Cell-Derived Schwann Cell-like Cells Spared Neuromuscular Junctions and Enhanced Functional Recovery After Peripheral Nerve Injury

**DOI:** 10.3390/cells13242137

**Published:** 2024-12-23

**Authors:** Yu Hwa Nam, Ji-Sup Kim, Yoonji Yum, Juhee Yoon, Hyeryung Song, Ho-Jin Kim, Jaeseung Lim, Saeyoung Park, Sung-Chul Jung

**Affiliations:** 1 Department of Biochemistry, College of Medicine, Ewha Womans University, Seoul 07804, Republic of Korea; queennnam@ewha.ac.kr (Y.H.N.); yjyum@ewha.ac.kr (Y.Y.); yjh6841@ewha.ac.kr (J.Y.); hrhssh@ewha.ac.kr (H.S.); 2 Department of Orthopaedic Surgery, College of Medicine, Seoul Hospital, Ewha Womans University, Seoul 07804, Republic of Korea; kkimjsno1@naver.com; 3 Cellatoz Therapeutics Inc., Seongnam 13487, Republic of Korea; hjkim@cellatozrx.com (H.-J.K.); jlim@cellatozrx.com (J.L.); 4 Graduate Program in System Health Science and Engineering, Ewha Womans University, Seoul 07804, Republic of Korea

**Keywords:** tonsil-derived mesenchymal stem cells, Schwann cell-like cells, neuronal regeneration-promoting cells, peripheral nerve injury, nerve transection, neurorrhaphy, fibrin glue, nerve regeneration, neuromuscular junction

## Abstract

In general, the nerve cells of the peripheral nervous system regenerate normally within a certain period after the physical damage of their axon. However, when peripheral nerves are transected by trauma or tissue extraction for cancer treatment, spontaneous nerve regeneration cannot occur. Therefore, it is necessary to perform microsurgery to connect the transected nerve directly or insert a nerve conduit to connect it. In this study, we applied human tonsillar mesenchymal stem cell (TMSC)-derived Schwann cell-like cells (TMSC-SCs) to facilitate nerve regeneration and prevent muscle atrophy after neurorrhaphy. The TMSC-SCs were manufactured in a good manufacturing practice facility and termed neuronal regeneration-promoting cells (NRPCs). A rat model of peripheral nerve injury (PNI) was generated and a mixture of NRPCs and fibrin glue was transplanted into the injured nerve after neurorrhaphy. The application of NRPCs and fibrin glue led to the efficient induction of sciatic nerve regeneration, with the sparing of gastrocnemius muscles and neuromuscular junctions. This sparing effect of NRPCs toward neuromuscular junctions might prevent muscle atrophy after neurorrhaphy. These results suggest that a mixture of NRPCs and fibrin glue may be a therapeutic candidate to enable peripheral nerve and muscle regeneration in the context of neurorrhaphy in patients with PNI.

## 1. Introduction

Peripheral nerve injury (PNI) can be associated with trauma, cancer, and iatrogenic lesions, resulting in neurological loss and functional or structural impairment. These conditions can result in partial or complete loss of motor and sensory functions, which can cause neuropathic pain, reducing the quality of life of these patients [1,2]. Complete nerve transection is the most severe form of PNI, with complete destruction of the innervation, perineurium, and epineurium. This injury offers no chance for spontaneous recovery without treatment, as it leads to rupture of the axon, myelin sheath, and connective tissue [3,4].

Three basic methods are available for the repair of transected nerves: neurorrhaphy, nerve grafting, and tubulization repair [5]. Among them, neurorrhaphy is the standard treatment option, whereas nerve grafting can be performed for nerve injuries with segmental defects [4]. Allogenic nerve conduits, which are human-derived tissues, pose a significant financial burden to patients, and although decellularization is essential to avoid immune rejection during the grafting of nerve conduits, decellularization using highly toxic surfactants can be harmful to the body if left in place [6]. After nerve repair, axons regenerate distally from the site of injury at a rate of less than 1 mm per day [7]. If nerve regeneration does not occur within a limited time, motor endplate loss ultimately leads to muscle atrophy, which is irreversible. Therefore, the best approach to treat peripheral nerve injury is to increase the rate of nerve regeneration within a short period.

Schwann cells (SCs) play an important role in nerve regeneration in several aspects such as degeneration, remyelination, and axonal growth. Research aimed at treating PNI by transplanting SCs is actively underway [8,9]. However, primary cultured SCs are limited in supply and their mass culture is difficult; thus, research using SC-like cells differentiated from stem cells has been actively conducted recently [10,11,12]. Tonsil-derived mesenchymal stem cells (TMSCs) exhibit a high yield and proliferation rate and have the potential to differentiate into mesodermal, endodermal, and ectodermal lineages [13]. We have previously differentiated TMSCs into SC-like cells (TMSC-SCs) and used them for the treatment of PNI and peripheral neuropathies [14,15,16]. TMSC-SCs were termed neuronal regeneration-promoting cells (NRPCs) and were produced according to a standardized process at the Cellatoz Therapeutic good manufacturing practice (GMP) facility.

PNI leads to nerve damage as well as loss and functional impairment of target skeletal muscles [17]. It is known that the progression of neurodegenerative muscular atrophy involves a series of pathological events and complex molecular mechanisms that are still largely unknown [18]. Among them, hepatocyte growth factor (HGF) can promote peripheral nerve regeneration by activating repair SCs [19] and may also play an important role in neurogenic muscular atrophy [20]. HGF has also been reported to be used as a platform for developing therapeutics for neuromuscular diseases [21]. Therefore, we investigated the expression of HGF in NRPCs and treated peripheral nerves.

In this study, the transected nerves of PNI model rats were subjected to neurorrhaphy and then transplanted with a mixture of NRPCs and fibrin glue. Nerve regeneration, neuromuscular junction (NMJ) preservation, and muscle recoveries after treatment were observed using nerve conduction studies (NCSs), immunohistochemistry (IHC), transmission electron microscopy (TEM), and Western blotting (WB), the results of which were used to evaluate the PNI treatment efficiency.

## 2. Materials and Methods

### 2.1. Cultivation of TMSCs and NRPCs

TMSCs were isolated and cultured from tonsils collected from patients who underwent tonsillectomy. Informed written consent was obtained from all patients participating in this study. The study protocol was approved by the Ewha Womans University Medical Center institutional review board (IRB No. EUMC-2021-09-036). Isolated tonsillar tissues were rinsed with 1 × phosphate-buffered saline (PBS) three times and minced in Dulbecco’s Modified Eagle Medium (DMEM, Hyclone, Logan, UT, USA). Homogenized tissues were mixed with Collagenase type I (210 U/mL, Invitrogen, Carlsbad, CA, USA) and DNase I (10 μg/mL, Sigma-Aldrich, St. Louis, MO, USA). These mixtures were digested at 37 °C for 30 min. Digested tissues were filtered with a cell strainer (BD Biosciences, San Jose, CA, USA), and the cells were then spun down at 1000 rpm for 5 min. Pellets were resuspended in high-glucose DMEM containing 10% fetal bovine serum (FBS, Invitrogen, Carlsbad, CA, USA) and 1% penicillin–streptomycin (Sigma-Aldrich, St. Louis, MO, USA) and plated on 100 mm cell culture dishes. After 48 h, non-adherent cells were removed, and the remaining adherent cells were cultured in a DMEM growth medium. The cells were cultured in a humidified chamber under 5% CO_2_ in the air at 37 °C.

To induce the TMSCs to differentiate into NRPCs, 2 × 10^5^ cells/cm^2^ were plated in DMEM/F12 (Welgene Inc., Daegu, Republic of Korea) supplemented with 20 ng/mL epidermal growth factor (EGF, PeproTech, Cranbury, NJ, USA), 20 ng/mL basic fibroblast growth factor (bFGF, PeproTech, Cranbury, NJ, USA), and 2% B27 supplement (1:50, Gibco, Life Technologies, Burlington, ON, Canada). The cultures were replenished with fresh medium every 3–4 days for differentiating cells. After 7 days, neurospheres were triturated using a 25-gauge needle and replated in laminin-coated (Sigma-Aldrich, St. Louis, MO, USA) cell culture plates containing DMEM/F12 supplemented with 10% FBS, 10 ng/mL bFGF (PeproTech, Cranbury, NJ, USA), 5 ng/mL platelet-derived growth factor-AA (PDGF, PeproTech, Cranbury, NJ, USA), 14 µM forskolin (Sigma-Aldrich, St. Louis, MO, USA), and 200 ng/mL recombinant human heregulin-β1 (PeproTech, Cranbury, NJ, USA) for terminal differentiation. The cells were incubated for 10 days under these conditions, and then harvested for further investigations. NRPCs were stored in a vial with cryoprotectant (CryoStor^®^ CS10; CS10, BioLife Solutions, Bothell, WA, USA) and used for application immediately after thawing and produced according to a standardized production process at the Cellatoz Therapeutic facility (Seongnam, Republic of Korea). TMSCs and NRPCs used in this study were manufactured in a GMP facility. The characteristics of the NRPCs are presented in our previous paper [15].

### 2.2. Western Blot Analysis

The cells were washed with cold PBS and lysed in PRO-PREP Protein Extraction Solution (iNtRON Biotechnology, Seongnam, Republic of Korea) containing a 1 × phosphatase inhibitor cocktail solution (Dawinbio, Hanam, Korea) (PRO-PREP buffer) for 30 min on ice. The sciatic nerve tissue was rapidly frozen in liquid nitrogen and then homogenized in PRO-PREP buffer on ice for 30 min. These samples were centrifuged at 13,000 rpm for 10 min at 4 °C, and equal amounts of protein from the supernatant were separated by the TGX Stain-Free™ FastCast™ Acrylamide Kit (BIO-RAD, Berkeley, CA, USA) in 1 × Tris/Glycine/SDS Buffer (BIO-RAD, Berkeley, CA, USA) at 80 V for 2 h. Protein transfer was performed with Trans-Blot Turbo (BIO-RAD, Berkeley, CA, USA) onto polyvinylidene fluoride membranes (BIO-RAD, Berkeley, CA, USA) using protocol 1.5 mm gel for 10 min. The stained membranes were developed with Clarity Western ECL Substrate (BIO-RAD, Berkeley, CA, USA) and imaged with Amersham™ ImageQuant™ 800 (Cytiva Life Sciences, formerly GE Healthcare Life Sciences, Marlborough, MA, USA). The images were analyzed using ImageQuant TL (Version 10.0.261). Band intensities were quantified with Image J software (Version 1.49). The manufacturers and catalog numbers of antibodies used are provided in Table S1.

### 2.3. Treatment and Application

**Scheme 1:**
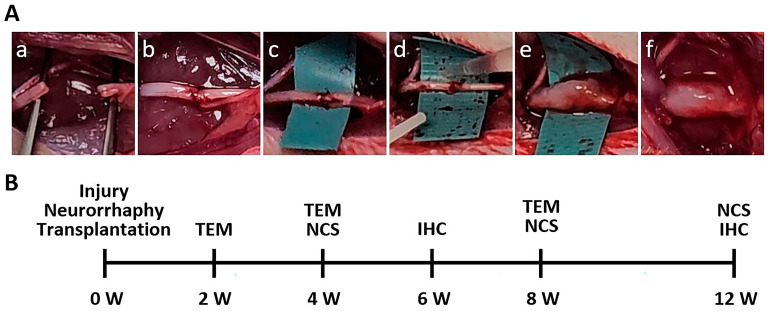
Experimental scheme of cell transplantation and schedule of the animal experiment. (**A**) After producing the PNI rat model via sciatic nerve transection (**a**), neurorrhaphy was performed on the transected nerve (**b**), the background material was placed under the nerve (**c**), and a mixture of fibrin glue and NRPCs was transplanted (**d**), wrapped with the background material (**e**) and hardened for 1 min; then, the background material was removed (**f**). (**B**) Schematic diagram of the experiment used for the assessment of efficacy after the application of NRPCs to PNI rats. PNI, peripheral nerve injury; NRPCs, neuronal regeneration-promoting cells; TEM, transmission electron microscopy; NCS, nerve conduction study; and IHC, immunohistochemistry.

The protocols used with the rats were approved by the institutional animal care and use committee of Ewha Womans University College of Medicine, and all experiments were performed in accordance with the approved guidelines and regulations (IACUC No. EUM20-049). The rats were housed in cages and maintained on a 12 h light–dark cycle with free access to food and water. Nerve transection surgery was performed on 7-week-old male Sprague–Dawley rats weighing 150–200 g under isoflurane inhalation anesthesia. To produce the PNI model rats, the sciatic nerves were cut with sharp microsurgical scissors. Neurorrhaphy was immediately performed on the transect nerve with two-needle sutures, and then the mixture of NRPCs in 100 μL CS10 and/or 200 μL fibrin glue (Baxter, Deerfield, IL, USA) was transplanted with background material (Keisei Medical Industrial, Japan), for which the detailed process is described in Scheme 1. Also, normal rats without nerve transection were used as a control group; the cell numbers for application and the experimental groups are described in Table 1. In addition, background materials were used for the injection of the mixture into the sciatic nerve, and the detailed process is described in Scheme 1A. Following the neurorrhaphy procedure, the sciatic nerve was sutured with 8–0 nylon suture and the skin was sutured with 3–0 silk suture (Ethilon, Ethicon, Somerville, NJ, USA).

### 2.4. Transmission Electron Microscopy

The sciatic nerves were biopsied at 2, 4, and 8 weeks after NRPC application and fixed with 2.5% glutaraldehyde in 0.1 M phosphate-buffered saline (pH 7.4) (Scheme 1B). The tissues were washed in 0.1 M phosphate-buffered saline and post-fixed with 1% osmium tetroxide in 0.1 M phosphate-buffered saline (pH 7.4) for 1 h, dehydrated with ethanol, and embedded in epoxy resin. Ultrathin sections, approximately 60–70 nm in thickness, were cut by an ultramicrotome (EMUC7, Leica, Deer Park, IL, USA) using a diamond knife. Sections were contrasted with 2% uranyl acetate followed by 0.25% lead citrate and observed with H-7650 TEM (Hitachi, Tokyo, Japan) at an accelerating voltage of 80 kV.

### 2.5. Nerve Conduction Study

The rats were used for NCS at 4, 8, and 12 weeks after NRPC application (Scheme 1B). The NCS was performed using a Nicolet VikingQuest apparatus (Natus Medical, San Carlos, CA, USA) and compound muscle action potential (CMAP) amplitudes and motor nerve conduction velocity (NCV) were determined. PNI rats were anesthetized with a 3% isoflurane (Hana Pharm, Seoul, Republic of Korea) in an air mixture on a 37 °C heating plate. The fur on the distal back and hind limbs was completely removed. Stimulating electrodes were placed on the biceps femoris to obtain a distal response and on the gluteus maximus to obtain a proximal response. The gap between the stimulating electrodes was 20 mm. The recording electrode was placed in the gastrocnemius muscle, and the reference electrode was placed in the Achilles tendon. Before stimulating and recording, all positions for needle electrodes were marked, and disposable subdermal needle electrodes (TE/S43-638, Technomed, Maastricht, The Netherlands) were used.

### 2.6. Immunohistochemistry

The sciatic nerves and gastrocnemius muscles of PNI rats at 6 and 12 weeks after NRPC application were fixed in 10% Neutral Buffered Formalin (BBC Biochemical, Mount Vernon, WA, USA) (Scheme 1B). The fixed tissues were embedded in paraffin and then sectioned into 5 μm-thick serial sections and placed onto microscope slides. The tissues were deparaffinized by incubation in xylene I and II (Duksan, Sejong, Republic of Korea) for 10 min each, followed by incubation in a graded ethanol series for 5 min each. The tissues were washed with 1 × PBS and permeabilized with 0.5% Triton X-100/PBS for 20 min at room temperature. Next, the tissues were placed in a blocking buffer containing 1% bovine serum albumin (BSA; Bovogen, East Keilor, VIC, Australia) and 0.5% Triton X-100/PBS for 1 h at room temperature. Subsequently, the tissues were incubated with the appropriate primary antibody overnight at 4 °C. After 3 washes in PBS, the tissues were incubated with the secondary antibodies for 1 h at room temperature. The stained tissues were mounted in VectaShield mounting medium containing 4′,6-diamidino-2-phenylindole (DAPI) (Vector Laboratories, Burlingame, CA, USA) and photographed using a fluorescence microscope (Ti2-U, Nikon, Tokyo, Japan) and slide scanner (VS200, Olympus, Tokyo, Japan). To confirm the NMJ, the gastrocnemius muscles of PNI rats were collected 6 weeks after NRPC application and fixed. The tissues were cryoprotected in 15% (6 h) and 30% (overnight) sucrose at 4 °C and then embedded in an optimum temperature medium (OCT; FSC 22 Frozen Section Media; Leica, Wetzlar, Germany) at −80 °C. Finally, the tissues were cryosectioned at a thickness of 40 μm and stained. A Z-stack of approximately 20 µm was captured using a confocal microscope (LSM800, ZEISS, Jena, Germany). The manufacturers and catalog numbers of the antibodies used here are provided in Table S1.

### 2.7. Hematoxylin and Eosin Staining

The rat gastrocnemius muscles were stained with hematoxylin and eosin (H&E) at 6 and 12 weeks after treatment for histological evaluation (Scheme 1B). Serial sections with a thickness of 5 μm were cut and stained using a standard technique to determine the histological changes in each group. The stained slides were observed and photographed under a microscope (BX51, Olympus, Tokyo, Japan).

### 2.8. Statistical Analysis

All experiments were performed at least 3 times. Statistical analyses were performed using Prism, version 10 (GraphPad Software, San Diego, CA, USA). The results are presented as the mean ± standard error of the mean (SEM). The statistical significance of TMSCs and NRPCs in WB was analyzed using Student’s *t*-test. To assess the significance of the differences in the observational results from TEM, NCS, WB, and IHC among the six groups, one-way ANOVA and Tukey’s post hoc multiple comparison test were used. Among the ultrastructural analyses, the “correlation between axon diameter and number of axons” was analyzed by two-way ANOVA and Holm–Šídák’s multiple comparison test. Significance was set at *p* < 0.05.

## 3. Results

### 3.1. Potential Characteristics of TMSCs and NRPCs for Peripheral Nerve Regeneration

**Figure 1:**
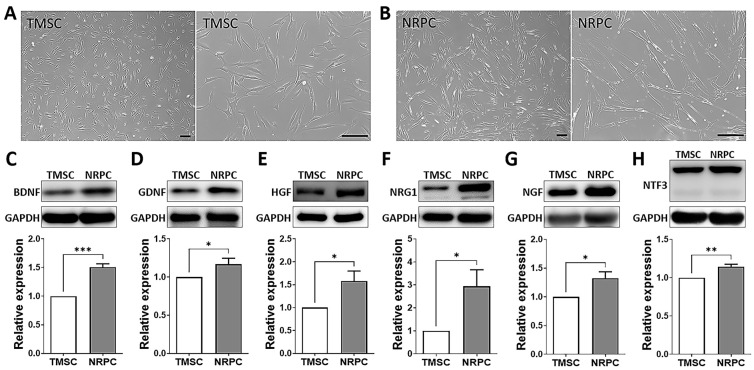
Differentiation of NRPCs derived from TMSCs and comparison of the expression of peripheral nerve regeneration-related markers. TMSCs (**A**) and NRPCs (**B**) were observed under an inverted microscope. The scale bars indicate 200 μm. Comparison of the expression of peripheral nerve regeneration-related markers, including BDNF (**C**), GDNF (**D**), HGF (**E**), NRG1 (**F**), NGF (**G**), and NTF3 (**H**), in TMSCs and NRPCs, as assessed by WB. The expression levels were analyzed quantitatively using Image J software (Version 1.49) and normalized to that of GAPDH. Student’s *t*-test was performed to analyze and compare TMSCs and NRPCs. The data are presented as the mean ± SEM of three independent experiments (* *p* < 0.05; ** *p* < 0.01; and *** *p* < 0.001). TMSCs, tonsil-derived mesenchymal stem cells; NRPCs, neuronal regeneration-promoting cells; WB, Western blotting; BDNF, brain-derived neurotrophic factor; GDNF, glial cell-derived neurotrophic factor; HGF, hepatocyte growth factor; NRG1, neuregulin 1; NGF, nerve growth factor; and NTF3, neurotrophin3.

A comparison of TMSCs and NRPCs revealed that TMSCs had a spindle-like fibroblast shape (Figure 1A), whereas NRPCs exhibited morphological changes after differentiation, with thinner cytoplasmic extensions, larger nuclei, and an elongated bipolar or tripolar spindle shape (Figure 1B). These cells were harvested and analyzed for the expression of neurotrophic factors at the protein level, including the brain-derived neurotrophic factor (BDNF), glial cell-derived neurotrophic factor (GDNF), HGF, neuregulin 1 (NRG1), nerve growth factor (NGF), and neurotrophic factor-3 (NTF3), using WB. The expression of each of these proteins was significantly increased in the NRPC vs. the TMSC group (Figure 1C–H). GDNF is involved in the proliferation, differentiation, and survival of nerve cells (Figure 1D), whereas HGF creates new blood vessels and promotes the growth and regeneration of nerves (Figure 1E). It was confirmed that the expression of BDNF and HGF was upregulated by about 1.5-fold in NRPCs compared with TMSCs (Figure 1C,E). In turn, the expression of NRG1, which promotes neuronal and glial responses during neuronal regeneration, was increased by about 3-fold in NRPCs compared with TMSCs (Figure 1F).

### 3.2. Restoration of Sciatic Nerve Structure After NRPC Application

**Figure 2:**
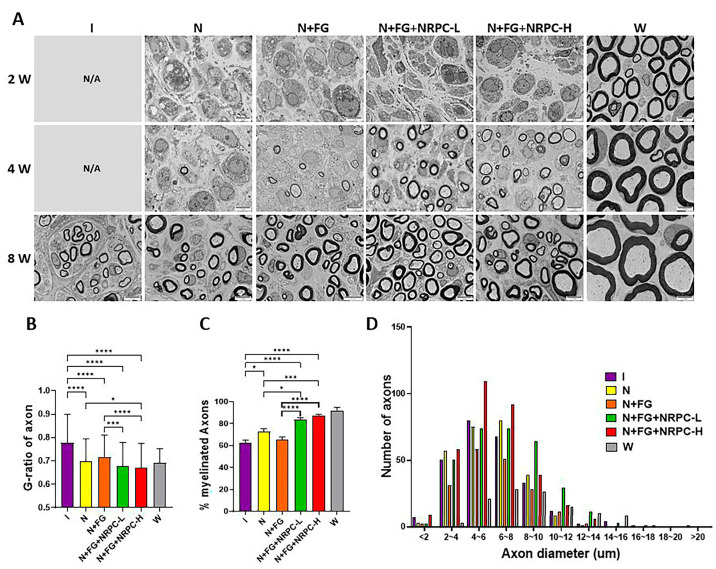
Observation of the myelination of the sciatic nerve of PNI rats using TEM. (**A**) Representative images of sciatic nerve cross-sections prepared at 2, 4, and 8 weeks after treatment (2 W, 4 W, and 8 W). The observation was not possible in the I group. Original magnification, 5000×. The axons in the only 8 W images were measured, followed by the quantification of the myelin thickness, indicating recovery of the sciatic nerve, based on the G-ratio (**B**) and the ratio of myelinated nerve fibers in the sciatic nerve (**C**). (**D**) Distribution of myelinated axons classified at 2 μm intervals between groups altered by NRPC application. Two-way ANOVA and Holm–Šídák’s multiple comparison test (row factor (number of axons per 2 μm diameter); column factor (group): (* *p* < 0.05; *** *p* < 0.001; and **** *p* < 0.0001)). PNI, peripheral nerve injury; TEM, transmission electron microscopy; NRPCs, neuronal regeneration-promoting cells; I, injury; N, neurorrhaphy; N + FG, neurorrhaphy + fibrin glue; N + FG + NRPC-L, neurorrhaphy + fibrin glue + NRPC-low; N + FG + NRPC-H, neurorrhaphy + fibrin glue + NRPC-high; W, wild type; and N/A, not applicable.

Two weeks after NRPC application, axons within the sciatic nerve were first observed using TEM (Scheme 1B); the observations were repeated at 4 and 8 weeks (Figure 2A). At this time point, a predominantly demyelinated pattern was observed in all PNI mouse groups, in contrast with the predominantly myelinated nerve fibers detected in the W group mice. In particular, the observation of axons in the I group was not possible after 2 and 4 weeks; therefore, photographs could not be acquired. In the representative images collected after 4 weeks, immature myelinated axons of a smaller size than that of the W group were observed very rarely in the negative control (N and N + FG) group and frequently in the transplant (N + FGNRPC-L and NRPC-H) group. The images obtained at 8 weeks showed observable myelinated axons in all groups (Figure 2A); these axons were measured and quantified. The G-ratio is the ratio of the inner axon diameter to the total outer myelin diameter, which is also known as the “axon/myelination ratio”. The G-ratio value was significantly highest in the I group among all groups, whereas the N + FG + NRPC-H group exhibited a clear decrease compared with all groups of the negative control (Figure 2B). Myelinated axons were significantly increased in the N + FG + NRPC-L and N + FG + NRPC-H groups compared with the negative control group, and displayed similar levels to those of the W group (Figure 2C). Furthermore, the distribution of myelinated axon diameters in PNI rats was analyzed by dividing them into 2 μm sections. The two transplant groups showed a larger number of myelinated axons compared with the negative control groups, and a similar trend in the distribution of axons larger than 10 μm to the W group (Figure 2D).

### 3.3. NRPC Application Led to the Recovery of Nerves

**Figure 3:**
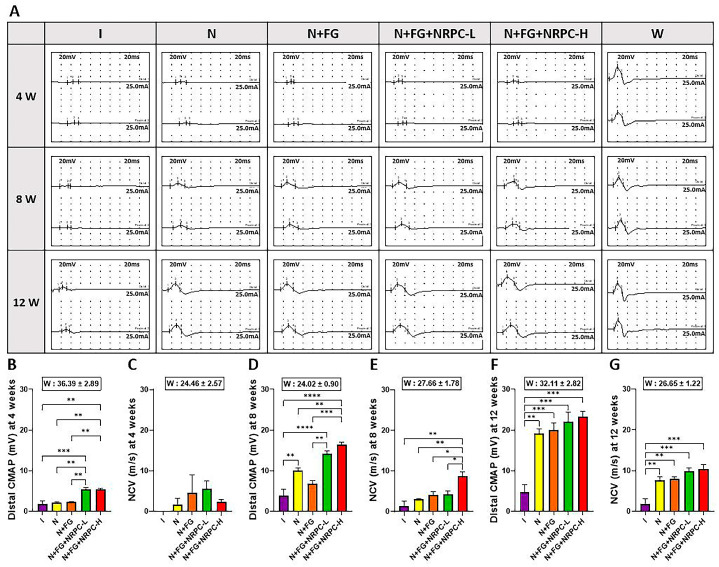
Confirmation of neural function recovery in PNI rats after NRPC application by NSC analysis. (**A**) Original graph of the NCS results at 4, 8, and 12 weeks (4 W, 8 W, and 12 W) after treatment. The distal CMAP and NCV values measured from the NCS results were quantified and compared between the groups at 4 W (**B**,**C**), 8 W (**D**,**E**), and 12 W (**F**,**G**), respectively. One-way ANOVA and Tukey’s post hoc test were performed for comparison between groups. The data are presented as the mean ± SEM (* *p* < 0.05; ** *p* < 0.01; *** *p* < 0.001; and **** *p* < 0.0001). PNI, peripheral nerve injury; NRPCs, neuronal regeneration-promoting cells; I, injury; N, neurorrhaphy; N + FG, neurorrhaphy + fibrin glue; N + FG + NRPC-L, neurorrhaphy + fibrin glue + NRPC-low; N + FG + NRPC-H, neurorrhaphy + fibrin glue + NRPC-high; W, wild type; NCS, nerve conduction study; CMAP, compound muscle action potential; and NCV, nerve conduction velocity.

NCS was performed and quantified at 4, 8, and 12 weeks after treatment; representative graphs for each experimental group are depicted in Figure 3A. CMAP was significantly increased in the N + FG + NRPC-L and N + FG + NRPC-H groups compared with the negative control groups (without cell application), whereas NCV showed no changes in any group at 4 weeks after treatment (Figure 3B). In week 8 after treatment, CMAP was increased in all groups compared with that observed in week 4; in particular, the N + FG + NRPC-H group exhibited a significant increase compared with all negative control groups (Figure 3D). The increase in CMAP suggests that the damaged motor nerves that control the muscles regenerated and that the corresponding damaged muscles were restored, at least to some extent. The decrease in NCV was associated with segmental demyelination and the loss of thick nerve fibers. At week 4, the NCV of group I was barely detectable, and the other groups showed no significant results (Figure 3C). However, at week 8 after treatment, the N + FG + NRPC-H group alone exhibited a significant increase compared with all other groups. These results indicate that the application of NRPCs promotes a faster rate of neural recovery (Figure 3E). At 12 weeks, CMAP and NCV were significantly increased in all groups compared with the I group (Figure 3F,G).

### 3.4. The Sciatic Nerve Regenerated After NRPC Application

**Figure 4:**
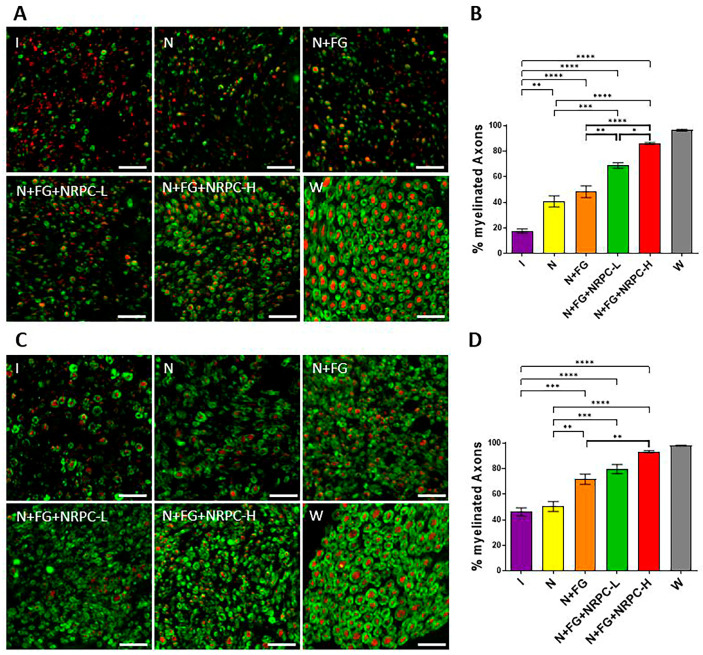
Axon regeneration and myelin formation in sciatic nerves in PNI rats treated with NRPCs. IHC staining for MBP (green) and NF-H (red) was performed at 6 (**A**) and 12 (**C**) weeks after NRPC application. The scale bars indicate 50 μm. Graph quantifying myelination of axons using IHC images at 6 (**B**) and 12 (**D**) weeks after NRPC application. One-way ANOVA and Tukey’s post hoc test were performed for comparison between groups. The data are presented as the mean ± SEM of three independent experiments (* *p* < 0.05; ** *p* < 0.01; *** *p* < 0.001; and **** *p* < 0.0001). PNI, peripheral nerve injury; NRPCs, neuronal regeneration-promoting cells; I, injury; N, neurorrhaphy; N + FG, neurorrhaphy + fibrin glue; N + FG + NRPC-L, neurorrhaphy + fibrin glue + NRPC-low; N + FG + NRPC-H, neurorrhaphy + fibrin glue + NRPC-high; W, wild type; IHC, immunohistochemistry; MBP, myelin basic protein; and NF-H, neurofilament heavy.

The sciatic nerves of the PNI rats were collected at 6 and 12 weeks after NRPC application. The improvement in myelination was evaluated based on the change in the expression of the myelin basic protein (MBP) and the neurofilament heavy (NF-H) protein, as assessed using IHC. In the W group, cross-sections of the green myelin surrounding the red nerves exhibited a round appearance, similar to donuts, both 6 and 12 weeks after treatment. The nerves of the PNI groups exhibited the best regeneration rate in the N + FG + NRPC-H group at 6 weeks after application, whereas almost no regeneration was observed in the I group (Figure 4A). In summary, the number of myelinated axons was the lowest in the I group compared with the remaining groups, and the N + FG + NRPC-H group had the highest number of myelinated axons compared with the other groups. In particular, the experimental group exhibited a significant increase in this parameter compared with the N + FG group among the negative control groups and showed a dose-dependent effect (Figure 4B). All PNI groups displayed a greater rate of overall nerve regeneration at 12 weeks after NRPC application compared with that observed at 6 weeks (Figure 4C). In addition, the number of myelinated axons in the I and N groups at 12 weeks after treatment was similar and lower than that recorded in the remaining groups. Moreover, the N + FG + NRPC-H group alone showed a significant increase in this number compared with the N + FG group to a level that was similar to that of the W group (Figure 4D).

### 3.5. The Gastrocnemius Muscle Recovered After NRPC Application

**Figure 5:**
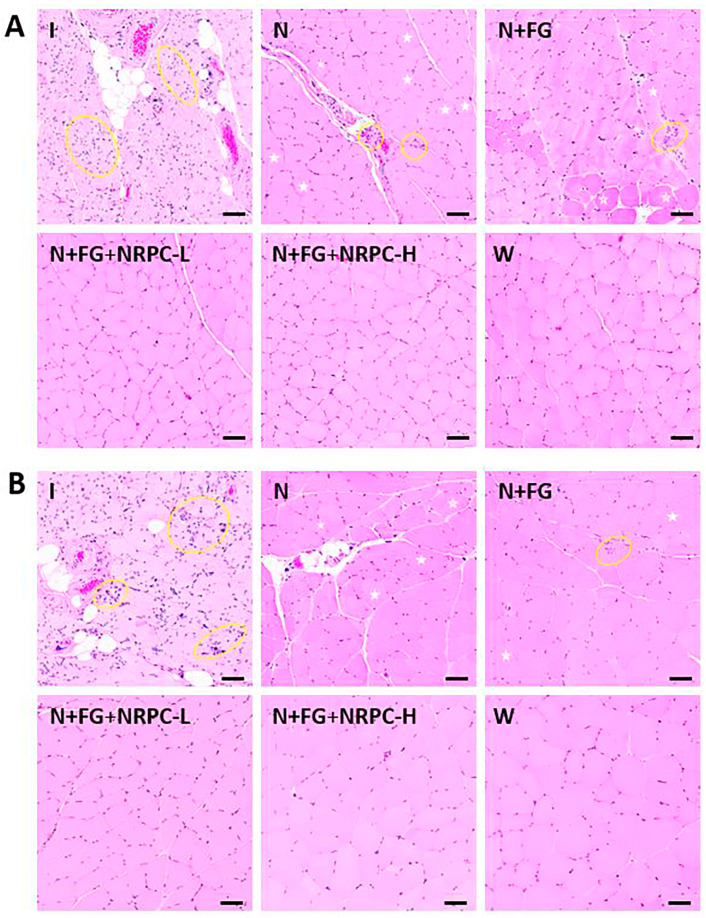
Confirmation of gastrocnemius muscle regeneration after NRPC application in PNI rats using H&E staining. Histological changes in the gastrocnemius muscle were determined at 6 (**A**) and 12 (**B**) weeks after treatment. The image of H&E staining was photographed under a microscope. White stars indicate big rounded fiber, and yellow circles indicate necrosis myofiber pattern. The scale bars indicate 50 μm. H&E, hematoxylin and eosin; NRPCs, neuronal regeneration-promoting cells; PNI, peripheral nerve injury; I, injury; N, neurorrhaphy; N + FG, neurorrhaphy + fibrin glue; N + FG + NRPC-L, neurorrhaphy + fibrin glue + NRPC-low; N + FG + NRPC-H, neurorrhaphy + fibrin glue + NRPC-high; and W, wild type.

The gastrocnemius muscles of rats were collected at 6 and 12 weeks after NRPC application and subjected to H&E and IHC staining. In images of the H&E staining, the necrotic myofiber pattern (yellow circles) presented in degenerative muscles was frequently observed in the I group but was not observed after NRPC application in the W, N + FG + NRPC-H, and N + FG + NRPC-L groups until 12 weeks after NRPC application (Figure 5). Intriguingly, the pattern was observed in the N and N + FG groups after 6 weeks (Figure 5A), and only in the N + FG group after 12 weeks (Figure 5B). Furthermore, big round fibers (white stars) were observed frequently after 6 weeks (Figure 5A), and rarely after 12 weeks, in the N and N + FG groups (Figure 5B). The W, N + FG + NRPC-H, and N + FG + NRPC-L groups displayed polygonal, regular muscle fiber shapes, and their size was slightly larger after 12 weeks (Figure 5B) than it was after 6 weeks (Figure 5A) of treatment, which is expected to be a change caused by the normal growth of the rats.

**Figure 6:**
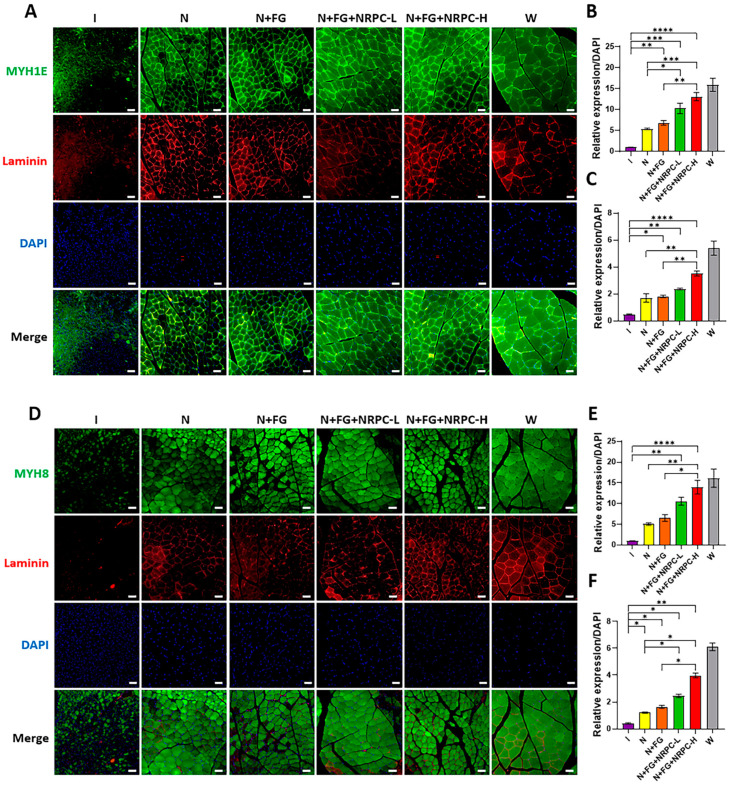
Confirmation of gastrocnemius regeneration in PNI rats at 12 weeks after NRPC application. Immunohistochemistry tissue staining for skeletal muscle-related makers, such as MYH1E (green), MYH8 (green), and laminin (red). The cross-sectioned slides of the muscles were double-stained with MYH1E and laminin (**A**) or MYH8 and laminin (**D**), and those images were photographed and merged. All cells were counterstained with DAPI (blue). The scale bars indicate 100 μm. The expression levels of MYH1E and laminin (**B**,**C**) or MYH8 and laminin (**E**,**F**) were compared by quantification with DAPI using Image J software (Version 1.49). One-way ANOVA and Tukey’s post hoc test were performed for comparison between groups. The data are presented as the mean ± SEM of three independent experiments (* *p* < 0.05; ** *p* < 0.01; *** *p* < 0.001; and **** *p* < 0.0001). PNI, peripheral nerve injury; NRPCs, neuronal regeneration-promoting cells; I, injury; N, neurorrhaphy; N + FG, neurorrhaphy + fibrin glue; N + FG + NRPC-L, neurorrhaphy + fibrin glue + NRPC-low; N + FG + NRPC-H, neurorrhaphy + fibrin glue + NRPC-high; W, wild type; MYH1E, myosin heavy chain 1 E; MYH8, myosin heavy chain 8; and DAPI, 4′,6-diamidino-2-phenylindole dihydrochloride.

The expression of skeletal muscle-related markers was evaluated and compared in the gastrocnemius muscles of each group harvested at 6 weeks (Figure S1) and 12 weeks (Figure 6) after NRPC transplantation by IHC. Myosin heavy chain 1 E (MYH1E), the most highly expressed protein detected in adult skeletal muscle, and laminin, which plays an important role in the normal contact between skeletal muscle and peripheral nerve axons, were selected as skeletal muscle-related markers. In the I group at both 6 and 12 weeks, no myofibrillar morphology was detected, and the expression of both proteins was the lowest. In the N and N + FG groups, although a muscle fiber morphology was confirmed, the expression level of the proteins was low, whereas in the N + FG + NRPC-L and N + FG + NRPC-H groups, the expression of both proteins increased simultaneously, and the staining pattern was similar to that of the W group (Figure 6A and Figure S1A). The results of image quantitation at 12 weeks after treatment showed that the expression levels of both MYH1E and laminin (Figure 6B,C) were the highest in the N + FG + NRPC-H group, and this group statistically significantly increased compared to all the negative control groups.

In addition, the expression of myosin heavy chain 8 (MYH8), which is a protein that is most abundant in perinatal skeletal muscle and is expressed in regenerating muscle and laminin was assessed. The I group exhibited the lowest expression of these proteins, and its muscle fibers were very small at both 6 and 12 weeks (Figure 6D and Figure S1B). With the exception of the I group, muscle fibers were confirmed in all groups; however, the expression levels of these proteins were low in the N and N + FG groups and increased in the N + FG + NRPC-L and N + FG + NRPC-H groups at 6 weeks (Figure S1B). MYH8 expression was increased in the N, N + FG, N + FG + NRPC-L, and N + FG + NRPC-H groups at 12 weeks after treatment, albeit not to the degree observed in the W group (Figure 6D). In the graph of quantitation at 12 weeks after treatment, both the MYH8 and laminin (Figure 6E,F) were the highest in the N + FG + NRPC-H group, and this group showed statistically significant increases compared with all negative controls. These results also indicate the recovery of the gastrocnemius muscle after sciatic nerve recovery, and the graph of quantitation showed a similar pattern to the ratio of myelinated axons in Figure 4D.

### 3.6. Neuromuscular Junction Preservation After NRPC Application

**Figure 7:**
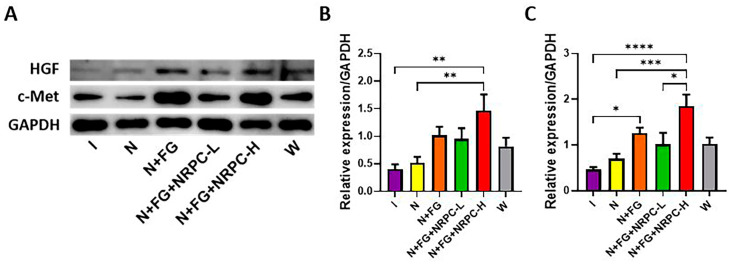
Expression of HGF and c-Met in PNI rat models by NRPC application. (**A**) Expression of HGF and c-Met in PNI rat nerves, as assessed by WB. The expression of HGF (**B**) and c-Met (**C**) was quantified using Image J software (Version 1.49), and its level was normalized to that of GAPDH. One-way ANOVA and Tukey’s post hoc test were performed for comparison between groups. The data are presented as the mean ± SEM (* *p* < 0.05; ** *p* < 0.01; *** *p* < 0.001; and **** *p* < 0.0001). HGF, hepatocyte growth factor; c-Met, cellular mesenchymal–epithelial transition factor; PNI, peripheral nerve injury; NRPCs, neuronal regeneration-promoting cells; WB, Western blotting; TMSCs, tonsil-derived mesenchymal stem cells; I, injury; N, neurorrhaphy; N + FG, neurorrhaphy + fibrin glue; N + FG + NRPC-L, neurorrhaphy + fibrin glue + NRPC-low; N + FG + NRPC-H, neurorrhaphy + fibrin glue + NRPC-high; and W, wild type.

HGF has been reported to be highly induced in damaged peripheral nerves and to activate the c-Met receptors present in Schwann cells and peripheral axons [22]. Moreover, HGF was confirmed in this study to be highly expressed after differentiation into NRPC (Figure 1D). The changes in the expression of HGF and c-Met, which can be considered one of the mechanisms of peripheral nerve regeneration by NRPCs, were confirmed by WB of rat sciatic nerve tissues collected at 6 weeks after treatment (Figure 7A). The expression of HGF was mostly increased in all groups but was statistically significantly increased only in the N + FG + NRPC-H group vs. the I and N groups (Figure 7B). The expression of c-Met was also significantly increased the most in the N + FG + NRPC-H group compared with the I, N, and L groups. However, the N + FG group also displayed a significant increase in the expression of this protein compared with the I group (Figure 7C).

**Figure 8:**
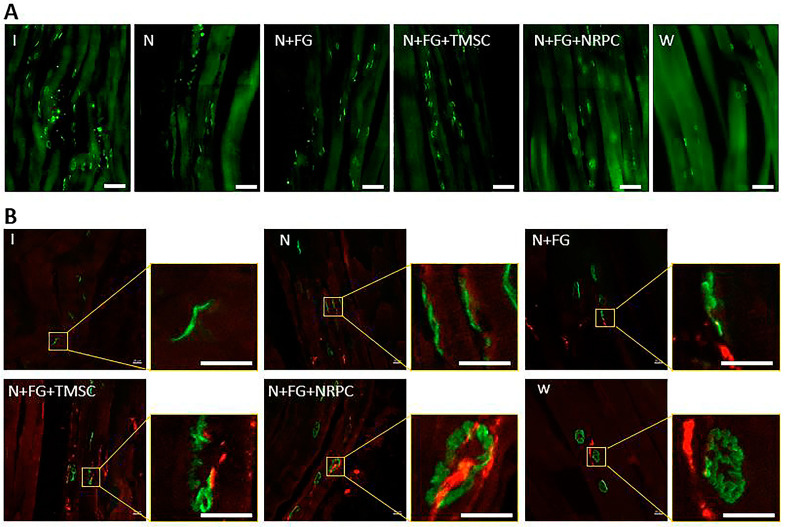
Neuromuscular junction analysis in PNI rat models by NRPC application. (**A**) To confirm α-BTX (green) expression in muscle endplates, the samples were cryosectioned, stained, and observed using a slide scanner. The scale bar represents 100 μm. (**B**) The morphology of NMJs was observed by α-BTX (green) and NF-H (red) expression at high magnification using a confocal microscope. The scale bar represents 20 μm. NRPCs, neuronal regeneration-promoting cells; PNI, peripheral nerve injury; IHC, immunohistochemistry; TMSCs, tonsil-derived mesenchymal stem cells; I, injury; N, neurorrhaphy; N + FG, neurorrhaphy + fibrin glue; N + FG + TMSC, neurorrhaphy + fibrin glue + TMSC; N + FG + NRPC, neurorrhaphy + fibrin glue + NRPC; W, wild type; α-BTX, alpha-bungarotoxin; NMJ, neuromuscular junctions; and NF-H, neurofilament heavy.

To further confirm these peripheral nerve regeneration mechanisms identified after NRPC application, we investigated the therapeutic effect through NMJ preservation more fundamentally. In this additional experiment, a high-dose (1 × 10^6^) undifferentiated TMSC (N + FG + TMSC) group was added, and the N + FG + NRPC-H (renamed as N + FG + NRPC in the additional experiment) group remained the transplant group. The dose of the N + FG + TMSC group used the number of cells tested in the preliminary study (Figure S3). The negative and positive control groups were identical to the original experimental groups, and two mice were used per group (Figure 8). Considering the peripheral nerve regeneration time confirmed at 6 weeks after treatment, as stated above, the rat gastrocnemius muscles were longitudinally cryosectioned, stained, and observed. The expression of alpha-bungarotoxin (α-BTX, green), which binds specifically to acetylcholine receptors (AchRs), was detected in muscle endplates. The N + FG + NRPC groups exhibited an intact shape, similar to that of the W group, whereas, in the I, N, and N + FG groups, the endplates displayed a shrunken and degenerated form. In the I group in particular, the muscle endplates were reduced in size and exhibited an irregular morphology, being either dispersed or fragmented (Figure 8A). The NMJ was stained for α-BTX (green) and NF-H (red) and observed using a confocal microscope. Connections between axons and muscle endplates were observed in the N + FG + NRPC groups, similar to the W group. In contrast, in the I, N, and N + FG groups, no axons connecting the endplates were visible (Figure 8B).

Furthermore, the endplates in the N + FG + NRPC groups exhibited a relatively normal pretzel-like structure, whereas only very few or no pretzel-like endplates were detected in the I, N, and N + FG groups. The NMJ morphology of the N + FG + TMSC group was more regenerated than that of the I, N, and N + FG groups, but was less regenerated than that of the N + FG + NRPC groups (Figure 8B).

## 4. Discussion

Two main treatment methods are available for PNI: surgical and non-surgical; however, a combination of surgical and non-surgical methods is usually employed. The non-surgical treatments include exercise [23], electrical stimulation [24], low-intensity pulsed ultrasound [25], and phototherapy [26]. In addition, other therapeutic modalities exist, such as growth factors [27], mesenchymal stem cells [28,29], secretome [30], gene therapy [31], nutritional therapy [32], and pharmacological methods [33]. In turn, the surgical methods include neurorrhaphy [4,5], autograft [34], and nerve allograft [35]. Moreover, recently, various materials have been studied to treat patients using nerve guide conduits [36,37]. Among the listed methods, microsurgical suture repair is the gold standard approach for the repair and reconstruction of peripheral nerves because it has traditionally provided the best results [38].

The use of sutures provides optimal tensile strength and durability after surgery; however, inflammation and fibroids may occur. Moreover, fibrin glue has been reported to also be effective in the repair of damaged peripheral nerves [5]. Nevertheless, although the use of fibrin glue alone can reduce inflammation, its low tension increases the likelihood of dehiscence. Therefore, better results have been obtained when the number of sutures is minimized and fibrin glue is used, which has the advantage of being safe for clinical application [39]. In this study, fibrin glue was used as a background material to help prevent inflammation and foreign substances and maintain the tensile strength. Therefore, it afforded the advantages of a precise realignment of nerve fibers during surgery and of shortening the surgical time. The fibrin glue used in this experiment was Tisseel, which contains added aprotinin, an antifibrinolytic component, to increase the duration of clots [40]. Moreover, it is a fibrin sealant that has been approved by the US Food and Drug Administration and is widely used in nerve repair [39,41]. In addition, the role of fibrin glue as a carrier has been well established in various studies [42,43,44]. In our preliminary study, the transplantation of fibrin glue and NRPCs as a mixture into PNI rats yielded a more improved effect than that observed for NRPC transplantation alone.

One critical factor that affects the clinical outcome of therapies for traumatic PNI is the time elapsed from the moment of injury to the surgical intervention. Conducting acute repair within 72 h after the injury can lead to favorable outcomes [45,46]. TMSCs are specialized to match the characteristics of the damaged tissue, are capable of providing specific signals and elements needed by that tissue, and can be easily cultured from allogeneic sources. Moreover, they can be rapidly deployed in situations requiring swift treatment [13]. This provides a significant advantage considering the importance of time in the initial treatment of nerve damage. TMSC-SCs (NRPCs) differentiated from TMSCs can be cultured from allogeneic sources (other individuals). This is a crucial consideration in the development of strategies for treating traumatic PNI. To date, autologous SC transplantation in humans after PNI has involved the harvesting of stem cells from the damaged nerve stump during the initial trauma surgery, their propagation, and the completion of nerve repair and SC transplantation within 30 days after the injury (from the traumatized sciatic nerve or sural nerve graft) [47]. In contrast, NRPCs are expected to be usable directly on the day of trauma in a frozen state.

The cell therapies for PNI offer two functions (i.e., accelerating axonal regeneration and preventing muscle atrophy by preserving the NMJs during the regeneration process) [48]. These functions hold significant importance for the clinical outcomes of peripheral nerve surgery. In particular, MSC therapy has been reported as one of the most innovative approaches in the field of nerve repair therapy because these cells have rapid self-proliferation and exhibit adrenocorticotropic hormone secretion/autocrine activity and differentiation potential [49]. Here, we confirmed that neurotrophic factors, such as HGF, NRG1, NGF, and NTF3, were highly expressed in NRPCs differentiated from TMSCs (Figure 1). This increase in neurotrophic factors from Schwann cells has a beneficial synergistic effect on the nerve repair process [50].

In addition, we demonstrated the occurrence of neuromuscular regeneration after NRPC application and selected HGF as the underlying mechanism. HGF plays an important role in motor, sensory, sympathetic, parasympathetic, and cortical nerve development, and c-Met is the sole receptor for HGF [22]. Recent studies have shown that HGF acts on neural stem cells to enhance neuronal regeneration and its application in the treatment of neurological disorders has been discussed [51,52]. We collected sciatic nerves from PNI rats and confirmed the expression levels of HGF and c-Met by WB. Both proteins were significantly upregulated in the N + FG + NRPC groups compared with the I and N groups (Figure 7B). PNI induces the expression of HGF and the activation of c-Met in the nerve crush mouse model [53]. Moreover, HGF/c-Met signaling is upregulated during muscle regeneration, promoting conversion to M2 macrophages and muscle regeneration [54]. Although the expression of HGF and c-Met in the N + FG group was similar to that observed in the N + FG + NRPC-L group, the correlation between fibrin glue and HGF/c-Met signals was not confirmed (Figure 7C). Consequently, treatment with NRPCs to manage PNI could potentially yield improved clinical outcomes, as NRPCs have the ability to enhance the expression of HGF within the sciatic nerve. In particular, the IHC results of the sciatic nerve (Figure 4) and gastrocnemius muscle (Figure 6) in this study also showed a statistically significant therapeutic effect in the N + FG + NRPC-H group compared to the N + FG group, and these results were similar to the expression patterns of the HGF and c-Met.

The NMJ connects a pre-synaptic axonal terminal to a post-synaptic region that is rich in AChRs, allowing the neuronal activation of muscle contraction. In murine animal models, the classic morphology of the NMJ is described as a pretzel-shaped structure [55]. Endplate re-innervation after nerve injury results in the degradation of functional AChRs and structural changes to the NMJ. In the I group, the NMJ appeared to be fragmented, whereas in the W group, it appeared as an intact pretzel. The N + FG + NRPC-H group also showed an intact pretzel morphology, similar to that of the W group (Figure 8B), indicating that the application of NRPCs prevented NMJ damage. Cell therapy not only accelerates axonal regeneration but also emphasizes the preservation of the NMJ, thereby minimizing muscle atrophy in the target muscle [48,56]. The time denervated is arguably the most important prognostic factor of recovery, at which point SCs are predicted to begin responding to the NMJ [57]. In clinical settings, it was generally reported that the muscle response to regenerating axons diminishes after 12 months post-nerve injury, thus failing to provide a meaningful recovery beyond this period [58]. We were able to accelerate nerve regeneration, prevent NMJ damage, and recover muscle injury via the application of NRPCs after neurorrhaphy. The reason for including the high-dose N + FG + TMSC group and excluding the N + FG + NRPC-L group in the NMJ analysis of this study was to prove that the therapeutic effect of NRPC administration was due to the NMJ regenerative factor secreted by TMSC-SC, not the anti-inflammatory function expected from undifferentiated TMSC administration, and because only the N + FG + NRPC-H group had a statistically confirmed therapeutic effect at the time of experimental groups were determined. This might be the first report on the recovery of denervated muscle atrophy in association with NMJ preservation applied by cell therapy in a PNI animal model with neurorrhaphy after peripheral nerve transection.

The success of cell therapies will rely on the development of solutions that address safety and immunogenicity, functional heterogeneity, the maintenance of biological activities, delivery challenges, and manufacturing challenges [59]. In particular, one of the concerns raised about stem cell therapy in clinical settings is its potential for malignancy. Although these concerns have not been completely alleviated, there were no side effects (such as malignancy) during the long-term follow-up of over 1 year in human cases with autologous SC transplantation for PNI [47,59]. In addition, the potential of allogenic NRPCs to treat CMT with no side effects has been reported [15], and no malignancies were observed in the clinical trials of NRPCs currently being carried out in patients with CMT. In conclusion, regeneration of both nerves and muscles was observed in the N + FG + NRPC groups and appeared to be dose-dependent in this study. Oncogenicity testing in a good laboratory practice institution will be performed when the clinical trial of this study begins in the future. By expanding the indications for diabetic neuropathy, spinal cord injury, and carpal tunnel syndrome, in addition to PNI caused by amputation, NRPCs promise a significant ripple effect as a treatment for peripheral nerve-related diseases.

## Figures and Tables

**Table 1:** Experimental groups of PNI rats used in NRPC application experiments.

Classification of Groups	Name of Groups	Neurorrhaphy	Fibrin Glue	Number of Cells	Vehicle (CS10)	Number of Animals
Negative control	I	-	-	-	-	15
Negative control	N	Yes	-	-	-	15
Negative control	N + FG	Yes	200 μL	-	100 μL	15
Transplant	N + FG + NRPC-L	Yes	200 μL	1 × 10^5^	100 μL	15
Transplant	N + FG + NRPC-H	Yes	200 μL	1 × 10^6^	100 μL	15
Positive control	W	-	-		-	15

I, injury; N, neurorrhaphy; N + FG, fibrin glue application after neurorrhaphy; N + FG + NRPC-L, mixed treatment of fibrin glue and low-dose NRPCs (1 × 10^5^) after neurorrhaphy; N + FG + NRPC-H, mixed treatment of fibrin glue and high-dose NRPCs (1 × 10^6^) after neurorrhaphy; and W, wild type.

## Data Availability

The data that support the findings of this study are available from the corresponding author upon reasonable request.

## Supplementary Materials

The following supporting information can be downloaded at: https://www.mdpi.com/article/10.3390/cells13242137/s1, Figure S1: Confirmation of gastrocnemius regeneration in PNI rats 6 weeks after NRPC application; Additional images for each group showing gastrocnemius muscle regeneration were confirmed after NRPC application to PNI mice by H&E staining; Figure S3. Preliminary study of NRPCs application in PNI rats; Table S1: Information on the antibodies used for immunohistochemistry and Western blotting.

## Abbreviations

The following abbreviations are used in this manuscript:

PNI	Peripheral nerve injury
SC	Schwann cell
TMSC	Tonsil-derived mesenchymal stem cell
TMSC-SC	SC-like cells differentiated from TMSC
NRPC	Neuronal regeneration-promoting cell
NCS	Nerve conduction study
IHC	Immunohistochemistry
TEM	Transmission electron microscopy
WB	Western blotting
PBS	Phosphate-buffered saline
DMEM	Dulbecco’s Modified Eagle Medium
FBS	Fetal bovine serum
EGF	Epidermal growth factor
bFGF	Basic fibroblast growth factor
PDGF	Platelet-derived growth factor
GMP	Good manufacturing practice
CMAP	Compound muscle action potential
NCV	Nerve conduction velocity
BSA	Bovine serum albumin
DAPI	4′,6-diamidino-2-phenylindole
H&E	Hematoxylin and eosin
SEM	Standard error of the mean
BDNF	Brain-derived neurotrophic factor
GDNF	Glial cell-derived neurotrophic factor
HGF	Hepatocyte growth factor
NRG1	Neuregulin 1
NGF	Nerve growth factor
NTF3	Neurotrophic factor-3
MBP	Myelin basic protein
NF-H	Neurofilament heavy
MYH1E	Myosin heavy chain 1 E
MYH8	Myosin heavy chain 8
NMJ	Neuromuscular junction
α-BTX	alpha-bungarotoxin
AchR	Acetylcholine receptor
