# Supplementary material for: Application of Mesenchymal Stem Cell-Derived Schwann Cell-like Cells Spared Neuromuscular Junctions and Enhanced Functional Recovery After Peripheral Nerve Injury
Source: Cells. 2024 Dec 23;13(24):2137. doi: 10.3390/cells13242137 (PMC11674609; doi:10.3390/cells13242137)
Supplement: Supplementary file 1 [file cells-13-02137-s001.zip › cells-3373039-supplementary.pdf]

cells

MDPI

Supplementary Materials

Application of Mesenchymal Stem Cell-Derived Schwann Cell-like Cells Spared Neuromuscular Junctions and Enhanced Functional Recovery After Peripheral Nerve Injury

Yu Hwa Nam [1,†], Ji-Sup Kim [2,†], Yoonji Yum [1], Juhee Yoon [1], Hyeryung Song [1], Ho-Jin Kim [3], Jaeseung Lim [3], Saeyoung Park [1,*] and Sung-Chul Jung [1,4,*]

[1] Department of Biochemistry, College of Medicine, Ewha Womans University, Seoul 07804, Republic of Korea; queennnam@ewha.ac.kr (Y.H.N.); yjyum@ewha.ac.kr (Y.Y.); yjh6841@ewha.ac.kr (J.Y.); hrhssh@ewha.ac.kr (H.S.)
[2] Department of Orthopaedic Surgery, College of Medicine, Seoul Hospital, Ewha Womans University, Seoul 07804, Republic of Korea; kkimjsno1@naver.com
[3] Cellatoz Therapeutics Inc., Seongnam 13487, Republic of Korea; hjkim@cellatozrx.com (H.-J.K.); jlim@cellatozrx.com (J.L.)
[4] Graduate Program in System Health Science and Engineering, Ewha Womans University, Seoul 07804, Republic of Korea
* Correspondence: saeyoung@ewha.ac.kr (S.P.); jungsc@ewha.ac.kr (S.-C.J.); Tel.: +82-2-6986-6197 (S.P.); +82-2-6986-6199 (S.-C.J.)
† These authors contributed equally to this work.

Table S1. Information on the antibodies used for immunohistochemistry and Western blotting.

Antibody	Host	Vendor	Catalog no.	Application
α-BTX, Alexa Fluor™ 488		Invitrogen	B13422	IHC
BDNF	Rabbit	Santa Cruz	sc-20981	WBC
c-Met	Rabbit	Abcam	ab51067	WBT
GAPDH	Rabbit	AbFrontier	LF-PA0212	WBC, WBT
GDNF	Goat	R&D Systems	AF-212-NA	WBC
Goat anti-chicken IgY (H+L) secondary antibody, Alexa Fluor 488	Goat	Invitrogen	A-11039	IHC
Goat anti-mouse IgG, IgM (H+L) secondary antibody, Alexa Fluor 488	Goat	Invitrogen	A-10680	IHC
Goat anti-rabbit IgG, HRP-linked Antibody	Goat	cell signaling	7074	WBC
Goat anti-rabbit IgG (H+L) cross-adsorbed secondary antibody, Alexa Fluor 568	Goat	Invitrogen	A-11011	IHC
HGF	Rabbit	Abcam	ab83760	WBC
HGF	Rabbit	Invitrogen	PA5-115354	WBT
Rabbit Anti-Goat IgG H&L HRP	Rabbit	Abcam	ab6741	WBC, WBT
Laminin	Rabbit	Novus Biologicals	NB300-144	IHC
MBP	Chicken	Sigma-Aldrich	AB9348	IHC
MYH1E	Mouse	DSHB	MF 20	IHC
MYH8	Mouse	DSHB	N3.36	IHC
NF-H	Rabbit	Santa Cruz	sc-20112	IHC
NGF	Rabbit	Abcam	ab52918	WBC
NRG1	Rabbit	Abcam	ab53104	WBC
NTF3	Rabbit	Abcam	ab6203	WBC

α-BTX: alpha-bungarotoxin; BDNF: brain-derived neurotrophic factor; c-Met: cellular mesenchymal–epithelial transition factor; GAPDH: glyceraldehyde 3-phosphate dehydrogenase; GDNF: glial cell-derived neurotrophic

factor; HRP: horseradish peroxidase; HGF: hepatocyte growth factor; IHC: immunohistochemistry; MBP: myelin basic protein; MYH1E: myosin heavy chain 1 E; MYH8: myosin heavy chain 8; NF-H: neurofilament heavy; NGF: nerve growth factor; NRG: neuregulin 1; NTF3: neurotrophin-3; WBC: Western blotting in cells; and WBT: Western blotting in tissue.

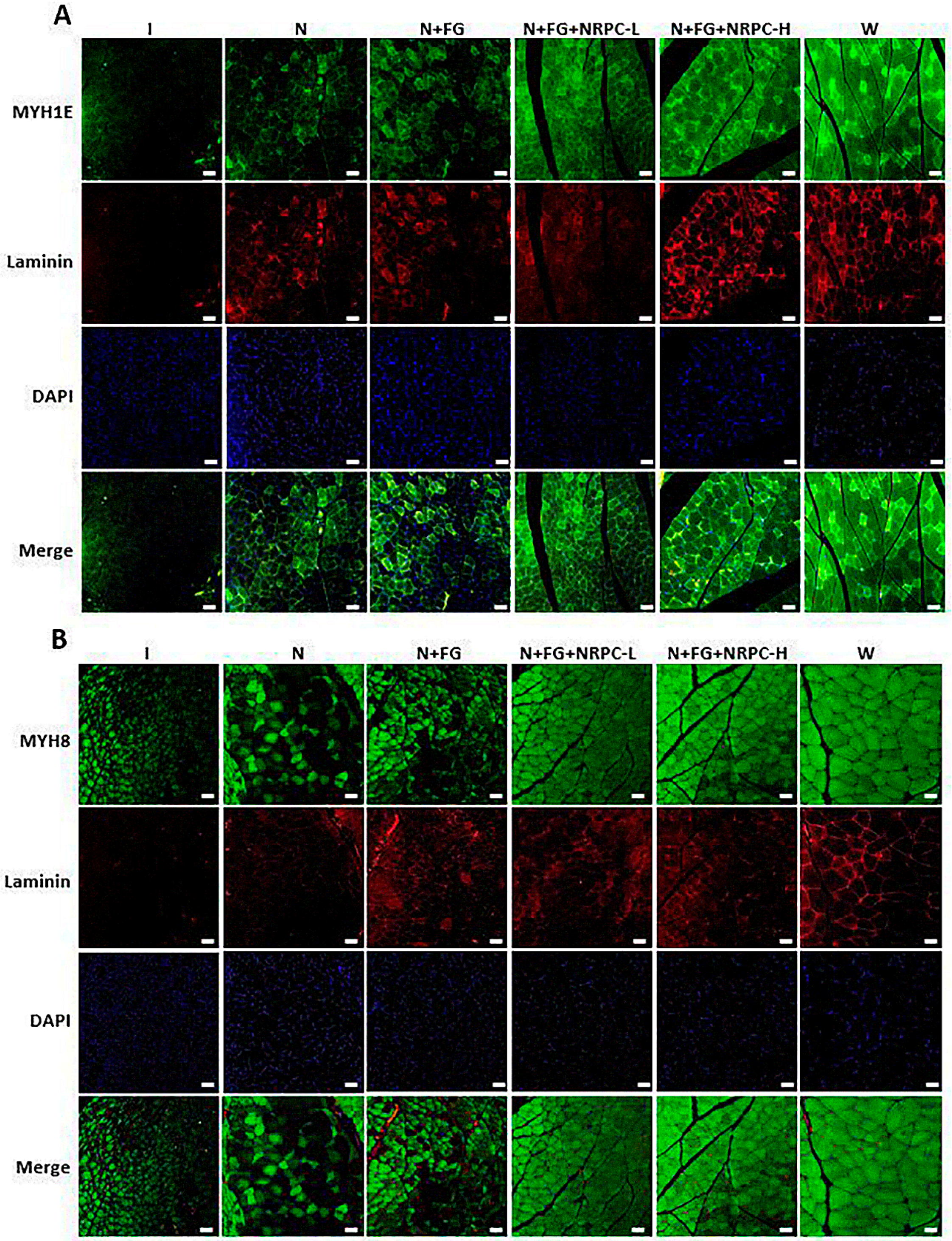

Supplementary Figure 1. Confirmation of gastrocnemius regeneration in PNI rats 6 weeks after NRPC application. Immunohistochemistry tissue staining for skeletal-muscle-related makers, such as MYH1E (green), MYH8 (green), and laminin (red). The cross-sectioned slides of the muscles were double-stained with MYH1E and laminin (A) or MYH8 and laminin (B), and the images were photographed and merged. All cells were counterstained with DAPI (blue). The scale bars indicate 100 µm. PNI, peripheral nerve injury; NRPCs, neuronal regeneration-promoting cells; I, injury; N, neurorrhaphy; N + FG, neurorrhaphy + fibrin glue; N + FG + NRPC-L, neurorrhaphy + fibrin glue +

NRPC-low; N + FG + NRPC-H, neurorrhaphy + fibrin glue + NRPC-high; W, wild type; MYH1E, myosin heavy chain 1 E; MYH8, myosin heavy chain 8; and DAPI, 4′,6-diamidino-2-phenylindole dihydrochloride.

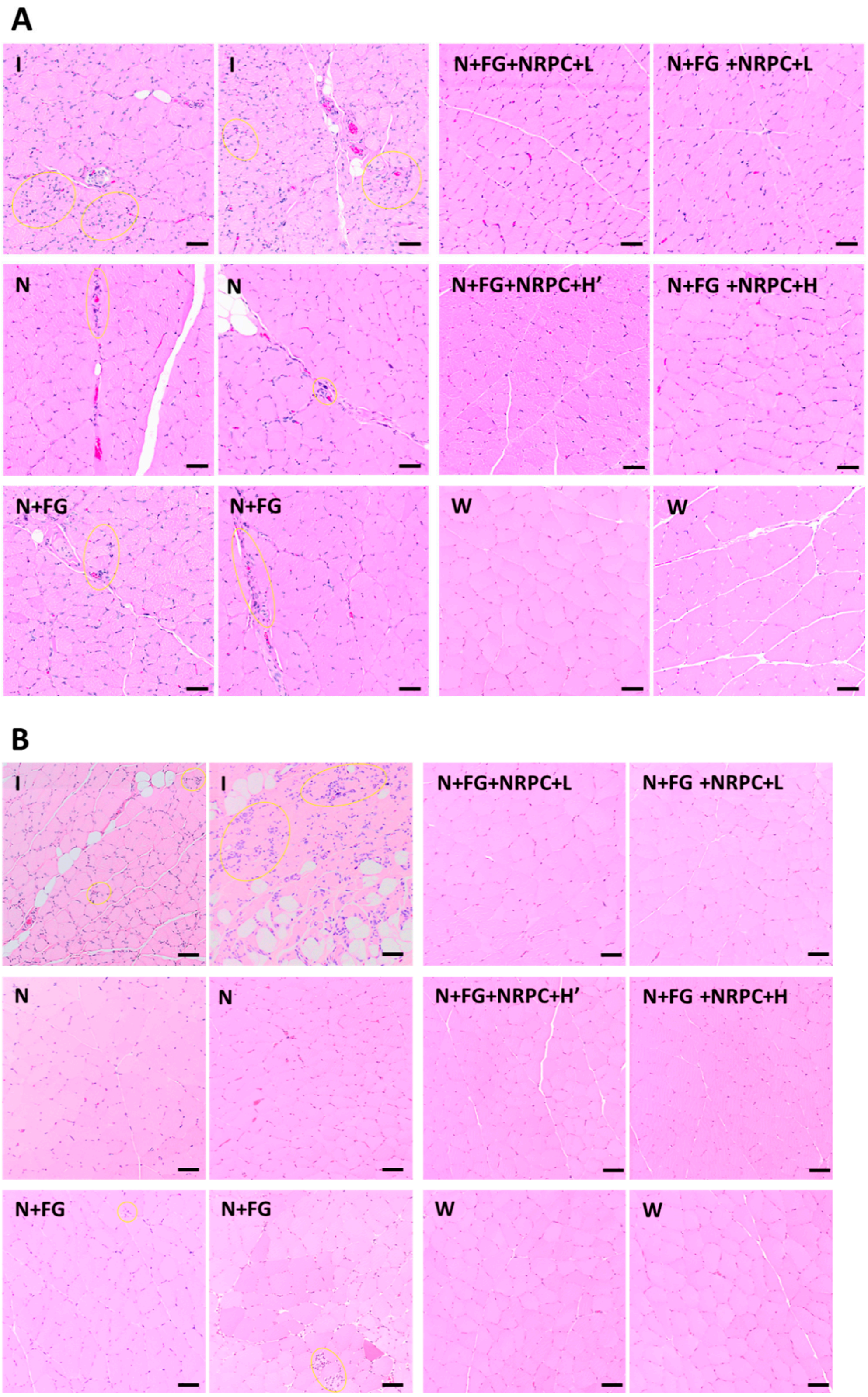

Supplementary Figure S2. Additional images for each group showing gastrocnemius muscle regeneration were confirmed after NRPC application to PNI mice by H&E staining. Histological changes in the gastrocnemius muscle were determined at 6 (**A**) and 12 (**B**) weeks after treatment. The image of H&E staining was photographed under a microscope. The scale bars indicate 50 µm.

O, necrosis myofiber pattern; H&E, hematoxylin and eosin; NRPCs, neuronal regeneration-promoting cells; PNI, peripheral nerve injury; I, injury; N, neurorrhaphy; N + FG, neurorrhaphy + fibrin glue; N + FG + NRPC-L, neurorrhaphy + fibrin glue + NRPC-low; N + FG + NRPC-H, neurorrhaphy + fibrin glue + NRPC-high; and W, wild type.

A

Classification of groups	Name of groups	Neurorrhaphy	Fibrin glue	Number of cells	Vehicle (CS10)	Number of Animals
Negative control	**N**	-	-	-	-	10
Negative control	**N+FG[1]**	Yes	200 µl	-	100 µl	10
Transplant	**N+FG+TMSC[2]**	Yes	200 µl	$1X10^6$	100 µl	10
Transplant	**N+FG+NRPC-L[3]**	Yes	200 µl	$1X10^5$	100 µl	10
Transplant	**N+FG+NRPC-H[4]**	Yes	200 µl	$1X10^6$	100 µl	10

N, Neurorrhaphy; [1]N + FG, Fibrin glue application after neurorrhaphy; [2]N + FG + TMSC, Mixed treatment of fibrin glue and high-dose TMSCs ($1{\times}10^6$) after neurorrhaphy; [3]N + FG + NRPC-L, Mixed treatment of fibrin glue and low-dose NRPCs ($1{\times}10^5$) after neurorrhaphy; [4]N + FG + NRPC-H, Mixed treatment of fibrin glue and high-dose NRPCs ($1{\times}10^6$) after neurorrhaphy.

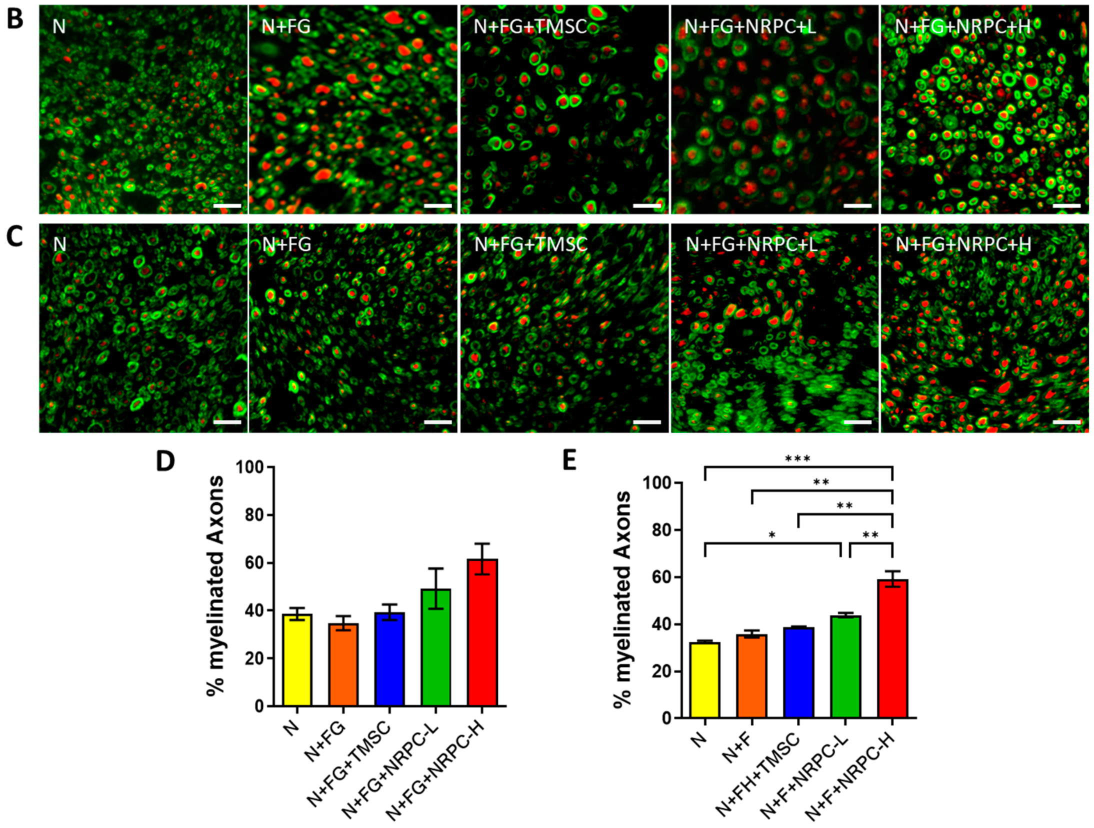

Supplementary Figure S3. Preliminary study of NRPC application in PNI rats. (**A**) Experimental groups of the rats used. Axon regeneration and myelin formation in sciatic nerves in PNI rats treated with NRPCs. IHC staining for MBP (green) and NF-H (red) was performed at 6 (**B**) and 12 (**C**) weeks after NRPC application. The scale bars indicate 25 µm. Graph quantifying myelination of axons using IHC images at 6 (**D**) and 12 (**E**) weeks after NRPC application. One-way ANOVA and Tukey's post hoc test were performed for comparison between groups. The data are presented as the mean ± SEM of three independent experiments (*$p < 0.05$; ** $p < 0.01$; and ***$p < 0.001$). PNI, peripheral nerve injury; NRPCs, neuronal regeneration-promoting cells; N, neurorrhaphy; N + FG, neurorrhaphy + fibrin glue; N + FG + TMSC, neurorrhaphy + fibrin glue + TMSC; N + FG + NRPC-L, neurorrhaphy + fibrin glue + NRPC-low; N + FG + NRPC-H, neurorrhaphy + fibrin glue + NRPC-high; IHC, immunohistochemistry; MBP, myelin basic protein; and NF-H, neurofilament heavy.
